# Evaluation of the p53 pathway in polycystic ovarian syndrome pathogenesis and apoptosis enhancement in human granulosa cells through transcriptome data analysis

**DOI:** 10.1038/s41598-023-38340-1

**Published:** 2023-07-19

**Authors:** M. Zanjirband, R. Hodayi, Z. Safaeinejad, M. H. Nasr-Esfahani, R. Ghaedi-Heydari

**Affiliations:** Department of Animal Biotechnology, Reproductive Biomedicine Research Center, Royan Institute for Biotechnology, ACECR, Isfahan, Iran

**Keywords:** Computational biology and bioinformatics, Genetics, Pathogenesis

## Abstract

The polycystic ovarian syndrome (PCOS) is closely associated with enhanced apoptosis of granulosa cells, which have a vital role in maturation of oocytes. p53 plays a critical role in the regulation of apoptosis and cell cycle arrest, metabolism and insulin resistance. The aim of this study was to investigate the impact of p53 pathway in enhancing apoptosis and abnormal function of granulosa cells. In this study, microarray analysis and RNA sequencing were downloaded from the GEO and used as datasets. Principal Component Analysis (PCA) and online SSizer tool were applied to evaluate the experiment quality control and sample sufficiency, respectively. Bioinformatics’ analyses were performed on the selected datasets, and validated by qRT-PCR and western blot analyses. Three datasets out of five ones were chosen for re-analyzing based on the PCA outcomes. 21 deregulated genes were identified via filters including *p* < 0*.*05 and |log2FC|≥ 1. Functional enrichment analysis confirmed the relevance of cell cycle regulation and apoptosis as common biological hallmarks in PCOS. Results have shown differentially expressed p53 target genes involved in apoptosis (*BAX, FAS, PMAIP1,* and *CASP8*), cell cycle (Cyclins, Cyclin dependent kinases), glucose metabolism and insulin resistance (*THBS1*), and p53 regulation (*MDM2*). Subsequently, the relative mRNA expression of *FAS*, *PMAIP1* and *MDM2* genes, and protein levels of p53 and MDM2 were confirmed using granulosa cells collected from 20 PCOS women and 18 control individuals by qRT-PCR and western blot, respectively. Results of this study represent the possible role of p53 pathway in pathogenesis of PCOS particularly, through the enhancement of apoptosis in granulosa cells.

## Introduction

The Polycystic Ovarian Syndrome (PCOS) is a heterogeneous multi-factorial endocrine and metabolic disease that impacts 3.4% of women worldwide based on the World Health Organization. PCOS is characterized by diverse clinical symptoms comprising reproductive dysfunction like chronic anovulation, polycystic ovary, infertility; endocrine and abnormal metabolism such as insulin resistance, dyslipidemia, obesity, hyper-androgenism and elevated luteinizing hormone (LH). In addition to genetics, lifestyle and environmental contributors are considered as risk factors associated with development of PCOS. According to the clinical symptoms and underlying etiology, the treatment strategy differs which includes treatment of ovulatory dysfunction and hyper-androgenism, improving insulin resistance and fertility, and lifestyle modifications^1^. Genomics, transcriptomics, proteomics and metabolomics studies have been performing with the aim of identifying the molecular pathways involved in the pathogenesis of PCOS. By way of example, RNA-sequencing (RNA-Seq) and microarray-based comparison of ovarian tissue from PCOS women with the controls revealed that Wnt (Wingless-related integration site) signaling pathway, insulin receptor signaling pathway, MAPK (Mitogen-Activated Protein Kinase) signaling pathway, aberrant status of mitochondrial energy metabolism, TNF (Tumor Necrosis Factor) signaling pathway, regulation of cell cycle and apoptosis are likely to be involved in the PCOS development^2–5^. However, according to our knowledge, the role of p53 pathway has never been investigated in this disease.

Granulosa cells (GCs), the critical somatic components of the ovary, play an essential role in supporting oocyte and provide a suitable microenvironment for follicular development and oocyte maturation. There are two major types of GCs named cumulus cells and mural GCs which surround the oocytes and the antrum, respectively. Cumulus cells are involved in providing nutrients to the oocyte and influencing the development of the oocyte whereas mural GCs have an endocrine function and support growth of the follicle^6^. Estrogens including estradiol and estrone are the key hormones produced by GCs following response to Follicle Stimulating Hormone (FSH) and diffusion of androgens from theca cells. GCs also produce disparate growth factors which interact with the oocyte during its development and impact the process of follicular growth^7^. Previous studies reported significantly increased apoptosis of GCs in PCOS individuals compared to those of the controls^8^. Hyper-androgenism affects GCs function and increases their apoptosis which are associated with abnormal folliculogenesis, decreased oocyte maturation and development of PCOS^9^.

The tumor suppressor p53, known as the guardian of genome, is a transcription factor located at the crossroad of a complex network of signaling pathways playing a key role in tumor suppression through inducing cell cycle arrest, senescence and apoptosis, and regulation of metabolism, immunity, inflammation and autophagy^10^. In addition, p53 is involved in the regulation of other cellular processes including diabetes and insulin resistance^11^, and steroid hormones regulation^12^. The present study aimed to recognize the possible role of p53 and its downstream target genes in development of PCOS. We hypothesized that comparisons of differentially expressed p53 target genes (DEp53TGs) in GCs taken from PCOS women versus controls would be helpful to identify p53 target genes involved in development of PCOS via promoting cell cycle arrest, apoptosis and abnormal function of GCs. To gain more benefits from multiple studies which provided dispersed pieces of knowledge on PCOS syndrome, functional enrichment analysis using EnrichR tool^13^ was also performed on the p53 target genes which were differentially expressed in PCOS subjects compared to controls. Lastly, qRT-PCR and western blot analyses were employed to verify some DEp53TGs involved in apoptosis.

## Results

**Figure 1:**
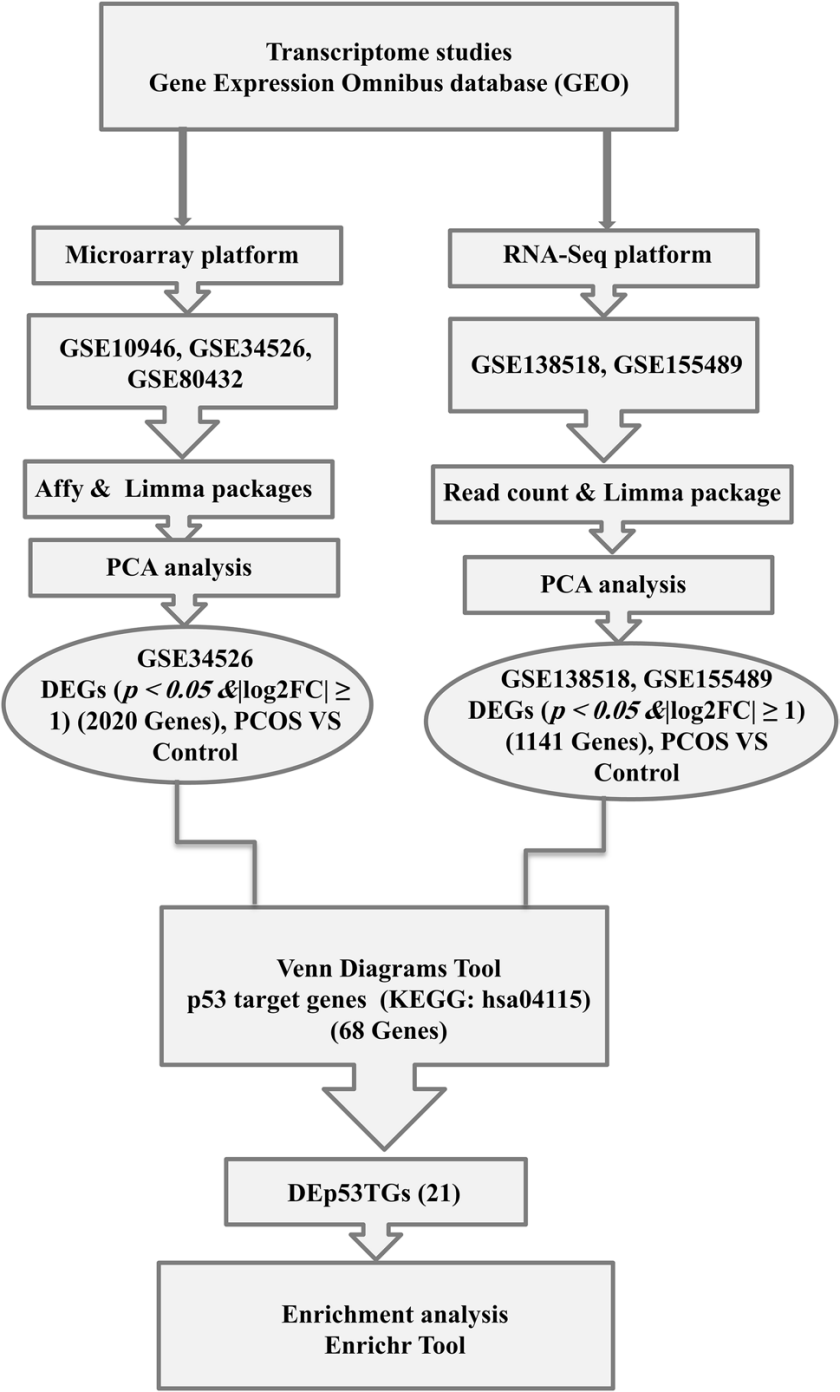
The flowchart of the bioinformatics strategy. *DEGs* differentially expressed genes; *DEp53TGs* differentially expressed p53 target genes.

In the present study, five mRNA expression datasets from GCs were reanalyzed to investigate DEp53TGs which might associate with development of PCOS through promoting apoptosis and cell cycle arrest, enhancing abnormal glucose metabolism and insulin resistance in GCs. For each dataset, two categories were compared including all PCOS individuals versus controls who were either healthy people or non-PCOS individuals (Supplementary Table 1). A normalized gene expression matrix including expressions levels of different genes in both PCOS and control samples was employed to make the PCA plots. For two datasets (GSE10946 and GSE80432), the samples are not separated between control and PCOS groups, so they were removed from this study (Supplementary Fig. 1). Overall, three datasets (GSE155489, GSE138518 and GSE34526) with a total number of 24 samples (Supplementary Table 1), 14 PCOS and 10 control samples, were selected for further analyses (Fig. 1).

### Differentially expressed p53 target genes (DEp53TGs) in GCs from PCOS women in comparison with controls

**Figure 2:**
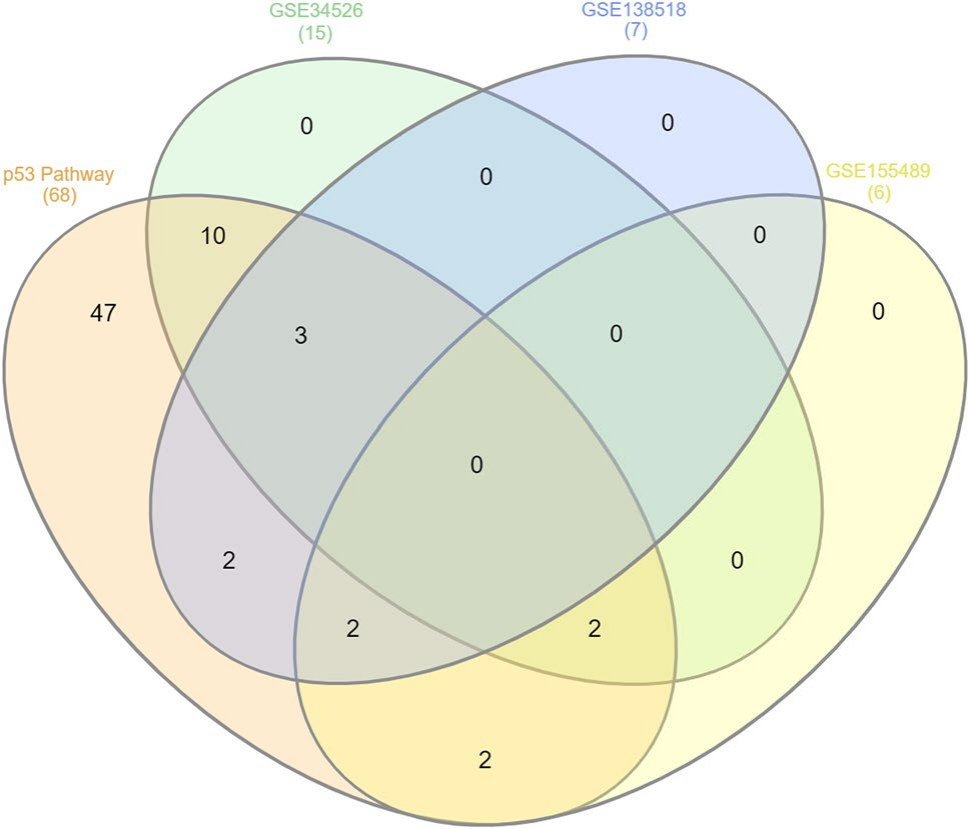
Venn diagram indicates the number of DEp53TGs (*p* < 0*.*05, |Log2FC|≥ 1) in each study, and the overlaps in respective comparisons. GSE34526 & p53 pathway: *BAX, BID, CASP8, CCNB2, CCNE2, CCNG1, MDM2, PTEN, SFN, STEAP3*; GSE138518 & p53 pathway: *ADGRB1, CCND1*; GSE155489 & p53 pathway: *GADD45G, SERPINE1*; GSE34526 & GSE138518 & p53 pathway: *CCNB1, CD82, FAS*; GSE34526 &GSE155489 & p53 pathway: *CCND3, THBS1*; GSE138518 & GSE155489 & p53 pathway: *CDK1, PMAIP1*.

When PCOS samples of each dataset were compared to control samples of the same dataset, a total of 21 genes were detected as DEp53TGs. The majority of up-regulated genes are involved either in induction of apoptosis including *BAX* (Bcl-2 Associated X-protein), *BID* (BH3 interacting-domain death agonist), *CASP8* (Caspase 8), *FAS* (Fas Cell Surface Death Receptor), *PMAIP1* (Phorbol-12-Myristate-13-Acetate-Induced Protein 1, known as *NOXA*), *PTEN* (Phosphatase and TENsin homolog), or the regulation of cell cycle arrest such as *CCNB2* (Cyclin B2), *CCND3* (Cyclin D3), *CCNE2* (Cyclin E2), *CDK1* (Cyclin Dependent Kinase 1), and *GADD45G* (Growth Arrest and DNA Damage Inducible Gamma). Interestingly, *MDM2* (Murine Double Minute 2), the major negative regulator of p53, was down-regulated which results in less proteasomal degradation of p53 and more transcriptional activity. Furthermore, several genes which have already proved to have impact on follicular atresia and glucose metabolism were also deregulated including *PTEN*, and *THBS1* (Thrombospondin 1) (Supplementary Table 2). Overall, there was consistency between the results observed in each dataset as 15 (71%) of DEp53TGs were up-regulated and 4 (19%) ones were down-regulated in PCOS women compared with their controls. However, inconsistent outcomes were observed in regard to *CD82* (Cell Differentiation 82) and *CCNB1* (10%) genes (*p* < 0*.*05, |log2FC|≥ 1, Supplementary Table 2). The Venn diagrams were constructed to represent the number of DEp53TGs in each comparison and the overlaps between the different comparison groups (Fig. 2). The numbers in overlapping parts of the shapes indicate the number of DEp53TGs in more than one study. Although the gene sets analyzed and the fold changes can be different between different platforms, seven DEp53TGs were statistically significant (*p* < 0*.*05) in more than one paired comparison comprising *THBS1* and *CCND3* (GSE34526 and GSE155489), *CDK1* and *PMAIP1* (GSE138518 and GSE155489), *CCNB1, CD82* and *FAS* ((GSE34526 and GSE138518) (Fig. 2).

**Figure 3:**
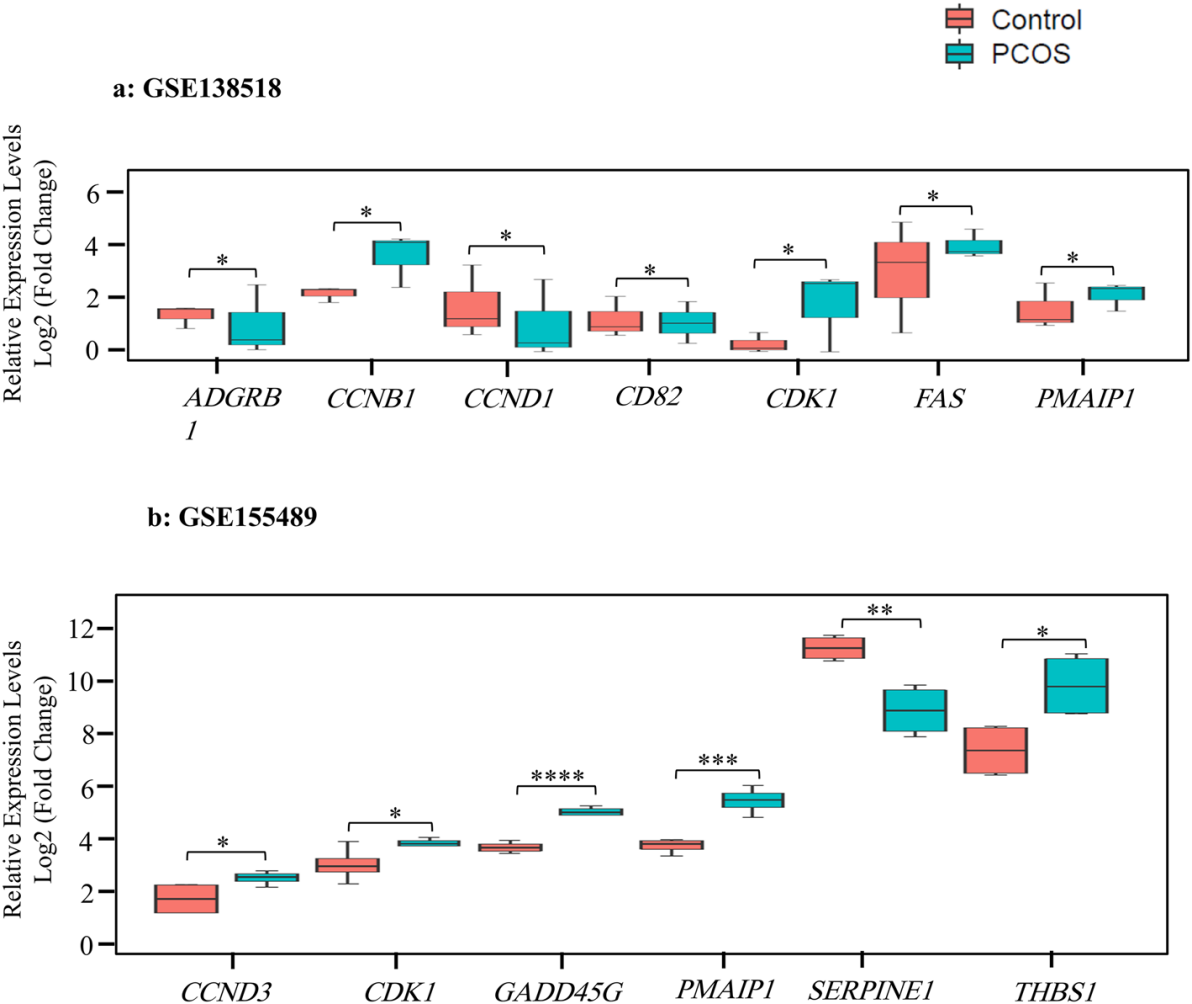
Expression levels of differentially expressed p53 target genes in 2 datasets. Box and whisker plots represent median and inter-quartile range (P25–P75). mRNA expression of the (**a**) 7-gene signature based on the GSE138518 dataset, (**b**) 6-gene signature according to the GSE155489 study. R package (version 4.1.2) limma, using an Empirical Bayes method, Benjamini–Hochberg adjustment for correction and control of the FDR (False Discovery Rate) across significant genes, and t-test, was applied to identify differentially expressed genes. *p < 0*.*05, **p < 0*.*01*,* ***p < 0*.*001*,* ****p < 0*.*0001*.*

**Figure 4:**
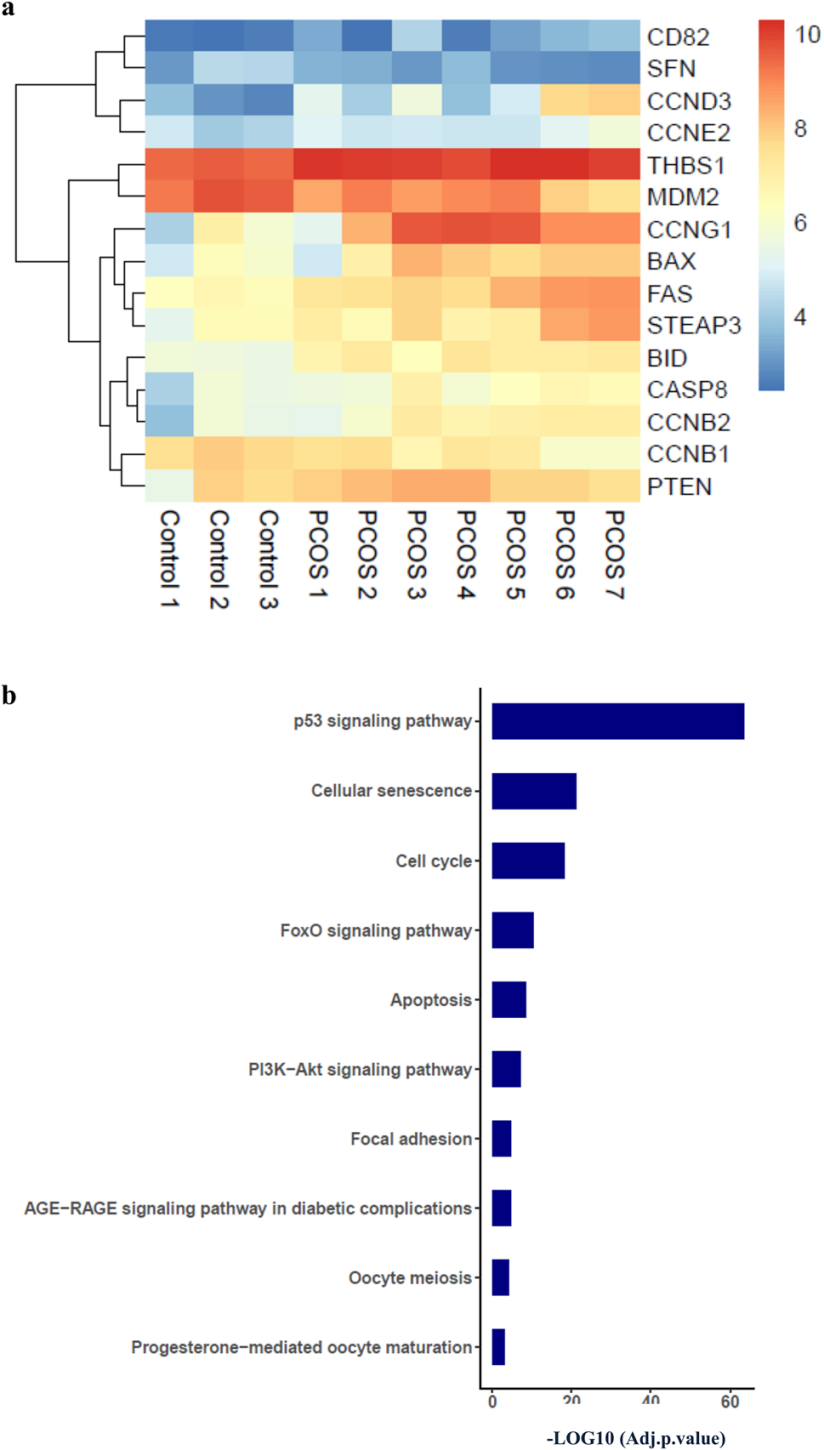
Differentially expressed p53 target genes in PCOS women compared to the control. (**a**) Heat-map showing the hierarchical clustering of differentially expressed p53 target genes in the GSE34526 dataset. (**b**) Significantly enriched KEGG pathways of associated PCOS related p53 downstream target genes which were differentially expressed (*p* < 0*.*05, |log2FC|≥ 1).

Two common inconsistent genes include *CCNB1* and *CD82* (GSE34526 and GSE138518). As summarized in Supplementary Table 2, *CD82* gene was down-regulated in PCOS individuals compared to the control in GSE138518 dataset but was up-regulated in GSE34526. In contrast to the *CD82* gene, *CCNB1* gene was up-regulated in PCOS individuals compared to the control in GSE138518 dataset but was down-regulated in GSE34526. Then, boxplots were employed to visualize DEp53TGs in GSE138518 (Fig. 3a) and GSE155489 (Fig. 3b) datasets. As the GSE34526 dataset has shown the highest number of DEp53TGs with the lowest *p*-value including 12 and 3 up- and down-regulated genes respectively, heatmap was drawn to visualize DEp53TGs on this dataset (*p* < 0*.*01, |log2FC|≥ 1). Hierarchical clustering analysis of these DEp53TGs indicated a significantly different gene expression pattern between PCOS and the control. The majority of genes were up-regulated in PCOS compared to the controls, which are involved in the enhancement of apoptosis and the regulation of cell cycle arrest (Fig. 4a).

### Functional analysis of differentially expressed genes

Based on the DEp53TGs, functional enrichment analysis was performed to identify the potential biological processes and pathways which might contribute to pathogenesis and development of PCOS. DEp53TGs in comparison between PCOS and control group mainly enriched in the p53 signaling pathway (21 genes) which was expected as we enriched DEp53TGs, cellular senescence (11 genes), cell cycle (10 genes), FoxO and PI3K-Akt signaling pathways (7 genes), apoptosis (6 genes), focal adhesion (5 genes), AGE-RAGE signaling pathway in diabetic complications and oocyte meiosis (4 genes) and progesterone-mediated oocyte maturation (3 genes) (adjusted *p value* < 0.001, Fig. 4b). The list of genes involved in these pathways are presented in the Supplementary Table 3. In accord with enriched signaling pathways, DEp53TGs were enriched in various biological processes including regulation of apoptotic process and cell cycle arrest (adjusted *p value* < 0.001, Table 1). About the cellular component and molecular function, those associated with apoptosis and cell cycle arrest were enriched comprising CD95 death-inducing signaling complex, mitochondrial outer membrane, and death-inducing signaling complex (adjusted *p value* < 0.01, Table 1). These enrichment analysis outcomes indicate that abnormal transcriptional alterations correlated with p53 signaling pathway may contribute to development of PCOS through enhancing apoptosis and dysregulation of cell cycle arrest in GCs.

**Table 1:** The functional analyses of deregulated genes targeted by p53 to identify the statistically significant GO terms.

Category/GO identifier	Terms	*p* Adj value	Overlapped genes
**GO_BP**^**a**^ GO:0000079	Regulation of cyclin-dependent protein serine/threonine kinase activity	4*.*00*E-*13	*CCNB2;CCND3;CCNB1;CCND2;CCND1;* *CCNE2;GADD45A;CCNG1;PTEN*
GO:1904029	Regulation of cyclin-dependent protein kinase activity	8*.*04*E-*13	*CCNB2;CCND3;CCNB1;CCND2;CCND1* *;CCNE2;GADD45A;CCNG1*
GO:0071900	Regulation of protein serine/threonine kinase activity	2*.*17*E-*10	*CCNB2;CCND3;CCNB1;CCND2;CCND1* *;CCNE2;GADD45A;CCNG1*
GO:0031571	Mitotic G1 DNA damage checkpoint signaling	2*.*80*E-*10	*CCNB1;CCND1;GADD45A;CDK1;MDM2;BAX*
GO:0044772	Mitotic cell cycle phase transition	4*.*36*E-*10	*STEAP3;CCND2;CASP8;GADD45A;IGFBP3;CDK1;* *PMAIP1;FAS;BAX;BID;THBS1;GADD45G*
GO:0042981	Regulation of apoptotic process	4*.*05*E-*09	*CCNB1;GADD45A;CDK1;MDM2;BAX*
GO:0006977 GO:1901992 GO:2000142 GO:0045787	DNA damage response, signal transduction by p53 class mediator resulting in cell cycle arrest Positive regulation of mitotic cell cycle phase transition Regulation of DNA-templated transcription, initiation Positive regulation of cell cycle	7*.*50*E-*09 8*.*16*E-*09 9*.*93*E-*09 1*.*32*E-*08	*CCNB1;GADD45A;CDK1;MDM2;BAX* *CCND3;CCNB1;CCND2;CCND1;CDK4;CDK1* *CCNB1;CCND1;CDK4;PTEN;CDK1;BAX* *CCND3;CCNB1;CCND2;CCND1;CDK4;MDM2*
**GO_CC**^**b**^ GO:0000307	Cyclin-dependent protein kinase holoenzyme complex	1*.*40*E-*18	*CCNB2;CCND3;CCNB1;CCND2;CCND1;* *CCNE2;CDK4;CCNG1;CDK1*
GO:1902554	Serine/threonine protein kinase complex	6*.*07*E-*18	*CCNB2;CCND3;CCNB1;CCND2;CCND1;CCNE2;CDK4;CCNG1;CDK1*
GO:0031265	CD95 death-inducing signaling complex	1*.*82*E-*04	*CASP8;FAS*
GO:0005741 GO:0005634	Mitochondrial outer membrane Nucleus	1*.*82*E-*04 1*.*82*E-0*4	*CASP8;PMAIP1;BAX;BID* *GADD45A;IGFBP3;PTEN;GADD45G;CCNB2;CCND3* *;CCNB1;CCND2;CCND1;CCNE2;CDK4;CCNG1;CDK1* *;MDM2;PMAIP1;BAX*
GO:0031968 GO:0031264 GO:0043231 **GO_MF**^**c**^ GO:0016538	Organelle outer membrane Death-inducing signaling complex Intracellular membrane-bounded organelle Cyclin-dependent protein serine/threonine kinase regulator activity	2*.*42*E-*04 2*.*91*E-*04 7*.*77*E-*04 6*.*64*E-*12	*CASP8;PMAIP1;BAX;BID* *CASP8;FAS* *GADD45A;IGFBP3;PTEN;GADD45G;CCNB2;CCND3* *;CCNB1;CCND2;CCND1;CCNE2;CDK4;CCNG1;* *CDK1;MDM2;PMAIP1;BAX* *CCNB2;CCND3;CCNB1;CCND2;CCND1;* *CCNE2;CCNG1*
GO:0019887 GO:0019900 GO:0008353 GO:0097472 GO:0004693	Protein kinase regulator activity Kinase binding RNA polymerase II CTD heptapeptide repeat kinase activity Cyclin-dependent protein kinase activity Cyclin-dependent protein serine/threonine kinase activity	1*.*15*E-*09 5*.*19*E-*04 0*.*001835336 0*.*004649277 0*.*004649277	*CCNB2;CCND3;CCNB1;CCND2;* *CCND1;CCNE2;CCNG1* *CCND3;CCNB1;CCND2;CCND1;* *GADD45A;FAS* *CDK4;CDK1* *CDK4;CDK1* *CDK4;CDK1*

a: GO_BP, Gene Ontology Biological Process (*p* < 0*.*001).

b: GO_CC, Gene Ontology Cellular Component (*p* < 0*.*01).

c: GO_MF, Gene Ontology Molecular Function (*p* < 0*.*01).

### Validation of differentially expressed FAS, PMAIP1 and MDM2 genes using qRT-PCR

To verify DEp53TGs explored via transcriptomic screening, some of DEp53TGs were selected for validation by q-RT PCR. A total of 20 PCOS women and 18 controls were recruited in the present study. The clinical features of the participants are presented in Table 2. There was a significant increase in the serum levels of AMH (Anti-Mullerian Hormone), LH (Luteinizing Hormone) and ratio of LH/FSH (Follicle Stimulating Hormone) in PCOS individuals compared to the controls.

**Table 2:** Clinical features of participants.

Variable^a^	Control (18)	PCOS (20)	*p-*value^c^
Age (years)	32.72 ± 1.10	31.71 ± 0.65	> 0*.*05
AMH (ng/ml)	3.48 ± 1.095	11.07 ± 3	** < 0*.*05**
BMI (kg/m^2^)	24.13 ± 0.85	26.58 ± 0.56	> 0*.*05
FBS (mg/dl)	99.11 ± 3.98	92.23 ± 3.54	> 0*.*05
FSH (mUI/ml)	5.25 ± 0.70	5.32 ± 0.6	> 0*.*05
LH (mUI/ml)	4.44 ± 0.74	7.57 ± 1.19	** < 0*.*0001**
LH/FSH ratio	0.87 ± 0.056	1.66 ± 0.24	** < 0*.*05**
PRL (ng/ml) ^b^	15.40 (1th 11.06, 3th 27.0)	16.40 (1th 10.63, 3th 28.0)	> 0*.*05
T3 (ng/ml)	97.05 ± 2.17	76.06 ± 25.03	> 0*.*05
T4 (ng/ml)	10.3 ± 1.53	8.04 ± 0.35	= 0*.*05
TSH (IU/L)	2.01 ± 0.27	2.54 ± 0.32	> 0*.*05
Vitamin D (ng/ml)	30.86 ± 5.40	32.61 ± 3.22	> 0*.*05

*AMH* Anti Mullerian Hormone, *BMI* Body Mass Index; *FBS* Fasting Blood Sugar; *FSH* Follicle Stimulating Hormone; *LH* Luteinizing Hormone; *PCOS* Polycystic Ovary Syndrome; *PRL* Prolactin; *T3* Triiodothyronine; *T4* Thyroxine; *TSH* Thyroid Stimulating Hormone.

a: With the exception of prolactin, data are presented as the mean ± standard error of mean (SEM).

b: Prolactin was a non-normally distributed variable and data are presented as the median, first (1th) and third (3th) quartiles.

c: Statistically significant *p* values shown as bold.

**Figure 5:**
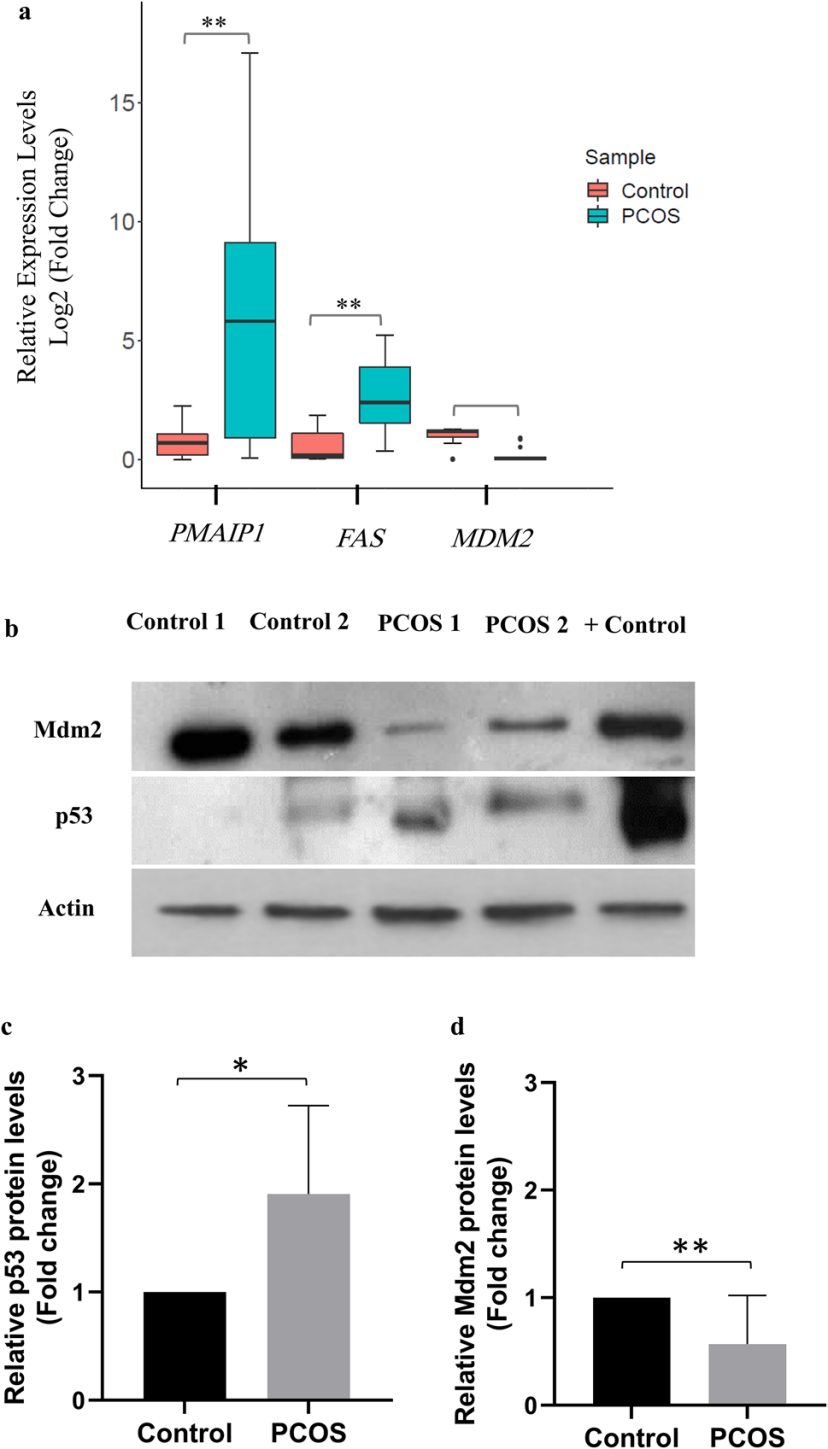
Validation of several differentially expressed p53 target genes. (**a**) qRT-PCR analysis of *PMAIP1, FAS* and *MDM2* genes in PCOS individuals (20) compared to the controls (18). The Mann–Whitney test was used and error bars represent SEM. (**b**) Western blot analysis of p53 and Mdm2 proteins in PCOS women compared to the controls. The Thermo scientific protein ladder, (10 to 180 kDa) was used as size standards for monitoring protein migration, protein transfer to membranes, and sizing proteins. The blots were cut into 3 pieces at ~ 70 kDa, a little under ~ 55 kDa, and a little under ~ 40 kDa for Mdm2 (90 kDa), p53 (53 kDa), and actin (42 kDa), respectively, prior to hybridization with antibodies. The intensity of p53 blots (**c**) (*p* < 0*.*05) and Mdm2 blots (**d**) (*p* < 0*.*01) for 6 PCOS and 6 control samples (western blot of the other 4 PCOS and 4 control samples are presented in Supplementary Fig. 2) was quantified by Image J software and normalized with the actin control. The blots were cropped and the original blots are presented in Supplementary Fig. 3. The samples derive from the same experiment and that blots were processed in parallel. The Student unpaired t-test was performed and error bars represent SEM. + Control, Nalm-6 wild-type *TP53* cell line treated with RG7388 (An inhibitor of p53-Mdm2 interaction). *, *p* < 0*.*05; **, *p* < 0*.*01.

Amongst DEp53TGs, *MDM2*, *FAS* and *PMAIP1* were chosen for proof (Fig. 5a). The criteria for selection of these genes are their key role on the enhancement of apoptosis and a high fold change value in more than one dataset (*FAS* and *PMAIP1*), being the main negative regulation of p53 (*MDM2*), and the relevance of the genes to future study of PCOS pathogenesis. Furthermore, intrinsic apoptotic pathway affecting mitochondrial function through the Bcl2 family members activation including *PMAIP1*, and extrinsic apoptotic pathway utilizing death receptors comprising *FAS* are two major signaling pathways for the development of apoptosis in GCs amongst the main three ones^14^. Besides, research has shown the importance of MDM2 protein in the impairment of oocyte maturation in the mice models as mice with MDM2 deficiency in GCs were infertile owing to the loss of oocyte maturation, ovulation, and fertilization. Conversely, mice with Mdm2/p53 double deletion in GCs indicated normal fertility, suggesting that p53-MDM2 axis in the ovarian GCs directs ovarian function by affecting their neighboring oocyte quality^15^. Thus, *MDM2* was selected as a crucial gene in this study and its expression levels were evaluated in both mRNA and protein levels.

Since distribution of aforementioned variables was non-normal distributed, the Mann–Whitney test was used to test whether or not the observed differences in regard to expression levels of each gene in two groups are statistically significant. The qRT-PCR results were consistent with the RNA-Seq and microarray data for all three genes as both *FAS* (by ∼10.4 fold, *p* < 0*.*01, median of control = 0.23 versus median of PCOS = 2.39) and *PMAIP1* (by ∼9.4 fold, *p* < 0*.*01, median of control = 0.62 versus median of PCOS = 5.82) were upregulated in PCOS samples compared to the controls, and the reverse was true for *MDM2* (by ∼15 fold, *p* < 0*.*0001, median of control = 1.20 versus median of PCOS = 0.08) (Fig. 5a).

### Validation of differential protein levels of p53 and MDM2 using Western blotting

For the controls and PCOS samples with sufficient amount of protein lysates for western blot, the protein levels of p53 and MDM2 were assessed. Interestingly, protein levels of p53 (by ∼1.9 fold, *p* < 0*.*05) and MDM2 (by ∼1.8 fold, *p* < 0*.*01) were significantly increased and reduced in PCOS samples compared to the controls, respectively (Fig. 5b–d). These results, consistent with bioinformatics analysis and qRT-PCR data, clearly indicate that protein levels of MDM2 significantly decreased in GCs of PCOS women in comparison to the controls, which consequently affects p53 levels and p53-mediated apoptosis pathway.

### Association between clinical or hormonal status of PCOS subjects and validated DEp53TGs

Spearman correlation test was used to evaluate the correlation of verified DEp53TGs including *FAS*, *MDM2* and *PMAIP1* to clinical and hormonal parameters of patients (Table 3). The correlation of the abovementioned validated genes with vitamin D was evaluated since Several studies reported that vitamin D regulates steroidogenesis in granulosa cells, vitamin D deficiency may enhance the risk of PCOS, and its overdose affects female reproduction function^16,17^. In regard to pro-apoptotic biomarkers which were significantly up-regulated in PCOS women, there was a significant positive correlation between expression levels of FAS with AMH (r = 0.63, *p* = 0*.*027), LH (r = 0.56, *p* = 0*.*023), prolactin (r = 0.52, *p* = 0*.*042) and vitamin D (r = 0.48, *p* = 0*.*034). However, over-expressed FAS showed a significant negative correlation with FSH (r =  − 0.56, *p* = 0*.*023) and the number of cumulus oocyte complex (COCs) (r =  − 0.53, *p* = 0*.*027). In line with *FAS* pro-apoptotic gene, up-regulated levels of *PMAIP1* in PCOS women indicated a significant direct correlation with AMH (r = 0.65, *p* = 0*.*014), LH (r = 0.50, *p* = 0*.*034), and vitamin D (r = 0.57, *p* = 0*.*012); and a significant reverse correlation with FSH (r =  − 0.52, *p* = 0*.*031) and the number of COCs (r =  − 0.57, *p* = 0*.*025). *MDM2*, the main negative regulator of p53, presented a significant negative correlation with 8 cells rates (r =  − 0.61, *p* = 0*.*01) and prolactin (r =  − 0.56, *p* = 0*.*035). As anticipated based on the function of *FAS* gene, there was a reverse correlation between the relative expression of this gene with blastocyst rates. Nonetheless, the correlation was close to be statistical significance (r =  − 0.41, *p* = 0*.*07).

**Table 3:** Spearman’s Rho correlation between relative expression levels of qRT-PCR validated genes and clinical, biochemical, and hormonal parameters in the GCs of subjects with PCOS.

Parameters	Genes^a^		
	*FA* Rho, *p value*	*MDM2* Rho, *p value*	*PMAIP1* Rho, *p value*
AMH	**0.63, 0.027**	0.53, 0.05	**0.65, 0.014**
FSH	− **0.56, 0.023**	0.027, 0.91	− **0.52, 0.031**
LH	**0.56, 0.023**	0.34, 0.17	**0.50, 0.034**
Prolactin	**0.52, 0.042**	− **0.56, 0.035**	− 0.22, 0.39
Vitamin D	**0.48, 0.034**	0.28, 0.29	**0.57, 0.012**
BMI	0.059, 0.80	− 0.195, 0.42	0.125, 0.60
FBS	0.063, 0.79	0.35, 0.16	0.047, 0.84
Blastocyst (%)	− 0.41, 0.07	− 0.41, 0.13	− 0.19, 0.45
COC	− **0.53, 0.027**	− 0.036, 0.90	− **0.57, 0.025**
Fertilization (%)	− 0.16, 0.5	− 0.43, 0.08	0.18, 0.44
GV (%)	0.19, 0.69	− 0.16, 0.57	0.22, 0.36
8 Cell (%)	0.19, 0.40	− **0.61, 0.01**	− 0.11, 0.68
Cleavage (%)	0.06, 0.79	0.17, 0.55	− 0.09, 0.72

*AMH* Anti Mullerian Hormone, *BMI* Body Mass Index; *COC* Cumulus Oocyte Complex; *FAS* Fas Cell Surface Death Receptor; *FBS* Fasting Blood Sugar; *FSH* Follicle Stimulating Hormone; *GV* Germinal Vesicle; *LH* Luteinizing Hormone; *MDM2* Murine Double Minute 2; *PMAIP1* Phorbol-12-Myristate-13-Acetate-Induced Protein 1.

a: Statistically significant correlations shown as bold.

## Discussion

Different studies have reported increased rate of apoptosis in GCs derived from PCOS women. Due to the crucial role of GCs in the maturation of follicle and developmental competency of the oocyte, identification of various mechanisms involved in their apoptosis would be helpful to find out the molecular pathways involved in development of PCOS^4,18,19^. The current study is the first report of systematically detecting DEp53TGs in GCs derived from PCOS women compared to the controls, and their potential roles in PCOS development.

GCs play major roles in supporting oocytes, providing essential nutrients and regulating oocyte maturation. In this regard, enhanced apoptosis and abnormal proliferation of GCs are reported during folliculogenesis in individuals with PCOS, accounting for anovulation and infertility^8,20–22^. Normal glucose metabolism in GCs is crucial, owing to their function for providing pyruvate and lactate as energy sources for oocytes. Therefore, insulin resistance in GCs is likely to influence their function and impair the potential of oocytes’ maturation^8^.p53 pathway has a vital role in various biological processes including cell cycle arrest and apoptosis^23–25^, metabolism and insulin resistance^10,11,26^, and steroid hormone regulation^12^. Hence, we hypothesized that DEp53TGs particularly; those involved in the cell cycle arrest and apoptosis might influence PCOS development. In this study, following constructing PCA plots in order to evaluate experiment quality control, DEp53TGs in GCs recognized from three mRNA expression datasets, microarray analysis (GSE34526) or RNA-Seq (GSE138518 and GSE155489), were applied for bioinformatics analysis to identify key genes, KEGG pathways and GO terms associated with PCOS, and finally some of DEp53TGs were validated using q-RT PCR and western blot analyses.

Comparison of the PCOS individuals with the group of controls yielded a number of p53 target genes that are differentially expressed. Amongst which, 21 genes (*p* < 0*.*05, |log2FC|≥ 1) were filtered and chosen for further analysis. Interestingly, in spite of using different platforms in these studies [GSE34526: GPL570 ( HG-U133_Plus_2) Affymetrix, GSE138518: GPL11154 Illumina HiSeq 2000, GSE155489: GPL20795 HiSeq X Ten] which can affects the gene sets analyzed and the fold changes^27^, seven DEp53TGs were statistically significant (*p* < 0*.*05) in more than one paired comparison indicating the importance of these DEp53TGs in PCOS compared with control.

21 DEp53TGs (*p value* < 0*.*05, |log2FC|≥ 1) were applied for bioinformatics analysis to identify KEGG pathways and GO terms associated with PCOS. Abnormal activity of these signaling pathways may contribute to pathogenesis or progression of diseases. Functional enrichment analysis revealed that DEp53TGs are densely interconnected in several pathways and cellular processes. Signaling pathways such as p53 pathway, cellular senescence, cell cycle arrest, FoxO signaling and apoptosis, were deregulated in the gene set. In regard to dysregulated p53 pathway, the majority of up-regulated p53 target genes enhances apoptosis pathway including *BAX*, *BID*, *CASP8*, *FAS*, *GADD45G*, *PMAIP1*, or regulates cell cycle progression such as *CCNB1*, *CCND3*, *CCNE2*, and *CDK1*. The most common up-regulated apoptotic genes are *BAX*, *FAS* and *PMAIP1*. *PMAIP1* encodes a pro-apoptotic member of Bcl-2 protein family with ability to displace Bak and Bim from their binding site on Mcl-1 and promote degradation of Mcl-1^28,29^. Bax and Bid, the members of the BCL-2 family, are involved in the induction of intrinsic apoptosis pathway^28^. Consistent with over expression of Bax observed in this study, a recent study reported significantly higher levels of Bax protein in GCs taken from PCOS individuals compared to the control which was associated with increased GCs apoptosis^30^. Other research indicated that over expression of Bax was associated with induced apoptosis in KGN granulosa cell line^31^ and mouse GCs^32^. *CASP8* encodes caspase 8 protein, a member of the cysteine-aspartic acid protease (caspase) family involved in apoptosis. It plays a key role in the apoptosis induced by Fas and other apoptotic stimuli^28,29^. Interestingly, both *FAS* and *CASP8* were simultaneously up-regulated in the reference study GSE34526 (Supplementary Table 1). Fas, a member of the TNF-receptor superfamily, enhances apoptosis through extrinsic apoptosis pathway^28,29^ considered as a major apoptosis signaling pathway in the ovarian follicle^14^. Some evidence indicates that PTEN^33^ and CD82^34,35^ proteins activate apoptosis as well. Besides, PTEN negatively regulates phosphatidylinositol 3 kinase (PI3K) and functions as an inhibitor of follicular activation in oocytes^36^. Follicular activation is a process by which primordial follicles in the ovary move from a quiescent to a growing phase. Since both transcriptional and non-transcriptional regulatory mechanisms are involved in apoptosis, and caspases are also regulated at the post-translational level by activating proteolytic cleavage, up-regulation of the pro-apoptotic and caspases genes could be indicative of the induced apoptosis. Considering the key functions of p53 in insulin resistance and regulation of steroid hormones, the activation of p53 induces insulin resistance through multiple tissues and organs. Studies indicated the importance of p53 over-expression in adipose tissue in enhancing insulin resistance. For example, insulin resistance and diabetes are developed via p53-mediated senescence of adipocytes and pancreatic beta cells, respectively, with the involvement of its downstream target genes such as increased expression of the cell cycle inhibitor p21/*CDKN1A*. p53 also regulates glucose transporters and insulin receptors^11,37^. Moreover, impairment of insulin action can be caused via p53-mediated PTEN overexpression in both insulin and non-insulin target tissues^37^. It is of note that PTEN was up-regulated in GSE34526 dataset used in this study. In regard to the regulation of steroid hormones, p53 plays a dual role in regulating both steroid hormone levels and their bioactivity. Research has shown that p53 transcriptionally regulates aromatase, a key enzyme converting androgens to estrogen, and SHBG (Sex Hormone Binding Globulin) expression as well^38^. Another study reported that MDM2-p53 pathway in GCs transcriptionally control a nuclear receptor steroidogenic factor 1 (SF1), a key regulator of ovarian function. More importantly, there is positive correlation of MDM2 and Sf1 levels in human GCs with the outcome of oocyte maturation and fertilization in patients undergoing infertility treatment^15^.

Cellular senescence and cell cycle pathways were recognized amongst the top 10 dysregulated pathways. Regulation of cell cycle is a very complicated process which is accurately controlled by a complex network of cyclins (Cyclins A, B, C, D, E, G), CDKs (Cdks 1, 2, 3, 4, 6) and CDKIs (The WAF family of CDKIs including p21^WAF1^, p27, p57, and the INK4 family comprising p15, p16, p18 and p19)^39,40^. Although periodic expression of classic cyclins such as cyclin A, B, D, and E, and the activity of Cdks are required for cell cycle regulation, the presence of cyclin G1 is also critical as its constitutive expression remains constant throughout the cell cycle. Cyclin G1 also plays a critical role in inactivating p53 through biochemical activation of MDM2, the main negative regulator of p53^39^. In regard to the reference study GSE138518, there was a significant increase in the expression levels of CDK1 and CCNB1 but CCND1 was significantly down-regulated. Although induced expression of CDK1 and CCNB1 enhance transition from G1 phase to S and onset of mitosis respectively, highly reduced expression of CCND1 (|Log2FC|= 1) leads to prevent transition from G0/G1 to S phase^39,40^. Overall, the comparison of all PCOS versus control, reveals the role of DEp53TGs in enhancing apoptosis, cellular senescence and cell cycle arrest of GCs, as well as reducing proliferation in GCs.

Another deregulated pathway in this gene set is FoxO signaling pathway, which participates in various physiological processes including cell proliferation, apoptosis, metabolism, inflammatory response; and oxidative stress resistance. Interestingly, in line with this study, the recently published research has demonstrated the association between FoxO pathway with PCOS. It was reported that FoxO1 expression, a member of FoxO subfamily expressed in almost all human tissues, was significantly elevated in cumulus cells of PCOS women compared to ones taken from non-PCOS individuals. It was also illustrated that FoxO1 has a possible role in the pathogenesis of PCOS through its role in regulating the gene expression, participating in gluconeogenesis, oxidative stress, cell proliferation and cell apoptosis^41^. These outcomes are consistent with this study indicating dysregulation of FoxO pathway in the GCs of PCOS subjects in comparison with those derived from control samples.

*MDM2*, known as *HDM2* in human, negatively regulates p53 through both binding to the N-terminus domain of p53 to inhibit its transcriptional activity and interacting with the DNA binding domain to promote its proteasomal degradation. Significantly decreased expression of MDM2 results in more stabilization and activation of p53, which consequently leads to increased cell cycle arrest and apoptosis^10^. *MDM2* was down-regulated in GCs in PCOS individuals compared to the control, and was commonly involved in the prominent pathways enriched in this gene set including cellular senescence, cell cycle, FoxO, and PI3k-Akt. A recent study has reported the impact of interaction between MDM2 and p53 in increased apoptosis and reduced proliferation in both KGN cells and primary GCs. The research has shown that MALAT1, an 8.7 kb long non-coding RNA, is down-regulated in PCOS GCs which results in enhancing their apoptosis. Normally, MALAT1 promotes the binding between p53 and MDM2 that further increases p53 proteasome degradation. Therefore, its down-regulation increases apoptosis in GCs through repressing p53 degradation^42^. In addition, research has demonstrated that low expression levels of MDM2 in the human granulosa cells leads to lower rates of oocyte maturation and fertilization, commonly observed in women with PCOS, indicating clinical relevance of the MDM2-p53 axis in ovarian granulosa cells in terms of human fertility^15^.

*THBS1* expression was consistently induced (GSE34526 and GSE155489) in the GCs derived from PCOS women in comparison to those taken from controls. Increased expression of THBS1 is in accordance with its potential role in insulin resistance, type 2 diabetes and dyslipidemia^43–45^, which are known to be involved in developing PCOS^1^. Importantly, research has also reported that THBS1 protein plays a key role in promoting follicular atresia and granulosa cell apoptosis^43,45,46^. The importance of *THBS1* in GCs has been reported as it is associated with decreased vascularity and proliferation of GCs in growing follicles^47^. Furthermore, *THBS1* might be involved in abnormal ovarian angiogenesis and destruction of abnormal follicles observed in PCOS syndrome^46,48^. With respect to the *SERPINE1* gene encoding a member of the serine proteinase inhibitor (serpin) superfamily, different studies reported its impact on various biological processes in GCs including glucose metabolism^49^, and lipopolysaccharide-induced porcine granulosa cell proliferation^50^.

In line with PCOS related pathways, the GO functional analysis of DEp53TGs indicated that GO terms are mainly associated with the regulation of cell cycle and apoptotic process. These findings suggest that theses dysregulated genes and pathways are likely to be associated with abnormal function of GCs and PCOS development.

Three main apoptosis signaling pathways are correlated with development of apoptosis in GCs comprising growth factors-, mitochondrial- and death receptors-induced apoptosis^14^. Owing to the significant upregulation of *PMAIP1* and *FAS* genes in this gene set and their key role in induction of mitochondrial- and death receptors FasL-Fas dependent apoptosis respectively, these genes were selected for validation. There was a significant rise in the expression levels of both genes in PCOS GCs based on the qRT-PCR results, indicating the potential role of those in enhanced apoptosis in GCs derived from PCOS women. Since *PMAIP1* and *FAS* are two p53 downstream target genes positively regulated by p53, their increased expression may account for the increase p53 activity in GCs of PCOS individuals. Interestingly, *MDM2* was down-regulated according to the bioinformatics analysis. Thus, it was also selected for validation, and both qRT-PCR and western blot analysis confirmed significantly decreased expression levels of MDM2 in PCOS GCs compared to control samples. Reduced MDM2 affects MDM2-p53 binding, which leads to repressing p53 degradation. Recently, Yan et al*.* have reported the impact of MDM2-p53 interaction in enhancing granulosa cell apoptosis in PCOS women^42^.

Remarkably, there was a significant negative correlation of *FAS* and *PMAIP1*expression with the number of COCs and early follicular phase serum FSH levels. Both of these genes are involved in enhancing apoptosis in different types of cells including GCs, which have critical roles in providing a suitable microenvironment for follicular development and oocyte maturation^14^. Therefore, increased expression levels of FAS and PMAIP1 might be a main cause of reduced number of COCs, despite higher number of COCs in PCOS women as compared to control individuals. For this discrepancy, see the explanation in the below discussion on the role of AMH in apoptosis in the below section. Several studies have reported that FSH acts as the primary survival factor during follicular atresia via playing an important role in decreasing apoptotic rate of porcine GCs^51^ and mouse GCs^52^. These studies give a clue to find out how the inverse correlation of pro-apoptotic genes *FAS* and *PMAIP1* with early follicular phase serum FSH levels influences the apoptotic rate of GCs and are consistent with correlation analysis in the PCOS group (Table 3). In regard to the observed positive correlation of *FAS* and *PMAIP1* with early follicular phase serum LH levels, LH has potential to protect cells against Fas-induced apoptosis as its protective effect has been reported in ovarian cancer HEY cells^53^. Despite anti-apoptotic role of LH, this study shows a positive correlation between LH and FAS expression. Again, this discrepancy could also be explained by the role of AMH in apoptosis (see the below discussion).

The positive correlation of pro-apoptotic genes *FAS* and *PMAIP1* with AMH is also notable. This correlation is consistent with the role of AMH in augmenting activation of caspase-3 and subsequent apoptosis reported in primary granulosa tumour cells and KGN cell line, a human granulosa tumour cell line^54^. Taken together, based on a recent study concluding that AMH in addition to regulating or restricting follicular activation, it also induces apoptosis by acting on small ovarian follicles to generate an upper limit to the size of the developing follicle pool before they become sensitive to FSH^55^. However, the results of this study in addition, indicate that in PCOS individuals, it is the FSH and LH level at this stage that determine the upper limits of the pool of follicle or number of COCs responding to ovarian stimulation. This means that on one hand AMH by inducing apoptosis is trying to limit the pool, while on the other hand FSH and especially increased in LH together are aiming to limit apoptosis. It appears that in this battle, AMH has not been able to limit the pool and this accounts for the limited number of COCs to undergo apoptosis and thereby accounts for higher number of COCs in the PCOS women as compared to control. It is interesting that such a correlation was not observed between FSH and LH with *FAS* and *PMAIP1*in the control group and this may account for the pathology of increased number of COCs in the PCOS individuals. How this difference is acquired remains to be explored. These observations are also consistent with numerous studies stating that in individuals with low AMH, it is the early follicular phase FSH level that determines the outcome of stimulation protocol in assisted reproductive cycles or the need for LH supplementation^56–58^.

Furthermore, there was a positive correlation between the aforementioned pro-apoptotic genes and vitamin D. These results are consistent with other studies demonstrated 1,25(OH)D3, the biologically active form of vitamin D, is capable of regulating the expression of an array of proteins involved in cell cycle arrest, such as p21, or apoptosis, including induction of the pro-apoptotic proteins and reduction of the anti-apoptotic molecules^59,60^. Although Masjedi and et al*.* reported that treatment of human PCOS GCs with vitamin D (100 nM, 48 h) augmented apoptosis rate in granulosa cells, no mechanism was found to clarify this effect^17^. The observed direct correlation of vitamin D serum levels with expression levels of FAS/PMAIP1 in PCOS GCs in this study is likely explained by the impact of vitamin D on induction of apoptosis in PCOS GCs. Therefore, vitamin D in PCOS individuals can increase rate of apoptosis in some follicles and limit the upper the size of the developing follicle pool and thereby the number of follicles which can be derived by internal FSH or by FSH stimulation. In addition, we also showed that hypervitaminosis D significantly increase the number of COCs retrieved in mice following FSH stimulation but the developmental competency of these COCs were severely jeopardized. The increase in the number of COCs was mechanistically attributed to the fact that on one side vitamin D promotes oocyte activation and maturation through down-regulation of AMHR-II receptors and, on the other hand, vitamin D inhibits oocyte activation and maturation through its suppressive action on AMH^16^. However, the latter effect that is “reduced developmental competency” may be explained by increased apoptosis induced in these follicles via vitamin D.

It is of note that the main limitation of the current study is the partially small number of individuals in each group for both bioinformatics analysis and primary GCs experiments. Although power analyses indicated the satisfaction of one type of indexes in regard to the sufficiency of sample sizes for the employed transcriptome dataset, increments in the sample sizes are strongly recommended to improve the efficiency of the statistical methods. Notably, it is generally due to use of small number of subjects in uploaded datasets in the GEO, which can be seen in other published studies in regard to DEGs in GCs. Among the strengths are expression profiling by high throughput sequencing, participation of homogenous groups of women with PCOS with age-matched healthy controls and validation of several Dep53TGs using two strong laboratory techniques, qRT-PCR and western blot.

In conclusion, comparison of the entire PCOS group with controls disclosed significantly differential expression of p53 downstream target genes involved in different cellular processes particularly, cell cycle progression, apoptosis, and follicle atresia. Verified deregulation of *FAS, PMAIP1* and *MDM2* genes via q-RT PCR; MDM2 and p53 proteins through western blot analysis indicate more evidence for the possible role of p53 pathway in pathogenesis of PCOS and how future intervention may reduce rate of p53-dependent apoptosis in these individuals.

## Materials and methods

### High-throughput gene expression datasets and their quality control

Raw data, five independently high throughput datasets, from microarray analysis (GSE10946, GSE34526 and GSE80432) and RNA-Seq (GSE138518 and GSE155489) in GCs including 34 PCOS samples and 29 controls (Table 1) were downloaded from the Gene Expression Omnibus database (GEO) (https://www.ncbi.nlm.nih.gov/geo/) ^18^. PCOS women participated in the abovementioned studies were diagnosed according to the Rotterdam revised criteria^61^. The raw data was utilized to reanalyze the gene expression profiles of GCs in PCOS individuals and controls, healthy people or non-PCOS patients. The Principal Component Analysis (PCA), an effective well-known methodology, and normalized gene expression matrixes were used for the experiment quality control^62^. Lastly, the online tool SSizer (https://idrblab.org/ssizer/) ^63^ was employed to assess the sample sufficiency for transcriptome data analysis (Selected cutoff of power value, area under ROC curve (AUC), accuracy, and overlap are 0.8, 0.9, 0.7 and 0.5 respectively).

### Identification of differentially expressed genes

The raw CEL microarray files were read into the Affy package of affylmGUI, a graphical user interface for the analysis of Affymetrix microarray data using the Linear Modes for MicroArray data (Limma) package^64^, for microarray analysis and read counts were employed as input for RNA-seq data. R package (version 4.1.2) limma was used in differentially expressed genes (DEGs) analysis for both RNA-seq and microarray studies^65^.

### Evaluation of the association between differentially expressed genes and p53 pathway

The Kyoto Encyclopedia of Genes and Genomes database (KEGG) (http://www.genome.jp/kegg/) was employed as pathway database to find the p53 pathway map (map04115) and a list of p53 signaling pathway genes (hsa04115)^66^. To investigate the role of p53 and its target genes in enhancing apoptosis and abnormal function of GCs, the InteractiVenn tool (www.interactivenn.net) was utilized to construct the Venn diagram^67^, which represents the number of DEGs in each comparison and the overlaps between the different comparison groups. DEp53TGs with *p* < 0*.*05 and |log2FC|≥ 1 in at least one of the comparisons are included.

### Functional enrichment analysis of differentially expressed p53 target genes

Enrichment analyses of DEp53TGs were performed using the Enrichr, a comprehensive gene set enrichment analysis web server (http://amp.pharm.mssm.edu/Enrichr)^68^. This powerful-web based tool contains a large collection of diverse gene set libraries, being available for analysis and download. In the current study, a list of DEp53TGs with *p* < 0*.*05 and |log2FC|≥ 1, 21 genes, (Supplementary Table 1) was used to perform enrichment analysis.

### Patient selection, sampling and cell isolation

The study was approved by the Ethics Committee of Royan Institute (IR.ACECR.ROYAN.R.EC.1401.027) and conducted in accordance with approved institutional guidelines. PCOS was defined according to the Rotterdam criteria^69^, which meets two of the following three features: oligo- or anovulation, clinical and/or biochemical signs of hyperandrogenism, and polycystic ovary by ultrasound. Informed consent was obtained from all women included in the study. All participants were candidates of IVF/ICSI (In Vitro Fertilization/Intra-cytoplasmic sperm injection) referring to the Isfahan Fertility and Infertility Center (Isfahan, IRAN) from January 2021 to July 2022. The inclusion criteria for the PCOS group were established diagnosis of PCOS based on the Rotterdam criteria and age between 18 to 40 years old. The control category comprised healthy women who were referring for family balancing and were not considered as PCOS bases on Rotterdam criteria. Individuals with congenital adrenal hyperplasia, androgen secreting tumors, Cushing's syndrome and endometriosis were not included in this study.

Following follicular puncture with the aid of vaginal ultrasound and collection of cumulus oocyte complex (COCs), the follicular fluid including GCs were collected from 20 PCOS women and 18 control individuals. The collected fluid was transported to the Royan Institute laboratory on ice and was centrifuged at 3300 RPM for 3 min at 25 °C and the supernatant was removed. Then, 4 ml of Tyrode’s solution (4 gr Sodium chloride, 0.1 gr Potassium chloride, 0.375 gr Magnesium chloride, 0.5 gr Sodium hydrogen carbonate, 0.025 gr Monosodium phosphate, 0.1 gr Glucose, 0.1 gr Calcium chloride, 500 ml distilled water) was added to the pellet and centrifuged at 1800 RPM for 8–10 min; and the supernatant was removed again. Then, 5 ml of RBC lysis (SUCROSE- Triton X100- MgCL2 1 M- TRIS HCL 0.5 M) was added to the pellet, incubated for 5 min at room temperature, centrifuged at 3000 RPM for 10 min at 25 °C, and the supernatant was disposed. The latter procedure was repeated. Finally, the pellet that contains GCs was used for the subsequent experiments^70^.

### Quantitative polymerase chain reaction (qPCR) validation

To validate several DEp53TGs involved in apoptosis, qRT-PCR was performed. Total RNA was extracted from GCs derived from PCOS women and control group using TRizol reagents (Yekta Tajhiz Azma, Iran) as per the manufacturer's recommendations. The quality of the RNA and its concentration was estimated with a NanoDrop ND-1000 Spectrophotometer (Thermo Fisher Scientific, U.S.A) by the ratio of 260 nm:280 nm. Total mRNA was converted to cDNA using the Ependorf AG 22331 Reverse Transcription System (Hamburg, Germany) as described by the manufacturer. Validated primers (Copenhagen, Denmark) are listed in the Supplementary Table 4. According to the manufacturer’s guidelines, SYBR® green RT-PCR master mix (Yekta Tajhiz Azma, Iran) was used to carry out qRT-PCR. The standard cycling parameters (Stage 1: 95˚C for 30 Sec, then 45 cycles of 95˚C for 5 Sec and 60˚C for 10 Sec and 72˚C for 30 Sec) on a sequence detection system (Thermo Fisher Scientific, Singapore) was employed to perform PCR reactions with 50 ng/μl of the cDNA samples per 10 μl final reaction volume. GAPDH was used as endogenous control for data normalization and data analysis was carried out using the ΔΔCt method.

### Western blotting

Protein levels of p53 and MDM2 were estimated using western blot analysis for the samples with sufficient amount of proteins. Total protein was extracted following TRIZOL isolation of nucleic acids from participants’ samples. Bradford solutions (100MG Coomassie Blue 250G, 50 ml ethanol 96%, 100 ml ortho-phosphoric acid 85% and bring volume to 1000 ml by adding distilled H2O) were used to estimate the concentration of protein in the cell lysates utilizing NanoDrop ND-1000 Spectrophotometer (Thermo Fisher Scientific, U.S.A).

Hand-poured gels, 8%, were prepared using Bio-Rad mini gel casting apparatus to separate proteins. The separated proteins were transferred by perpendicular electrophoresis to a nitrocellulose HybondTM C membrane (Amersham, Buckinghamshire, UK). The Thermo scientific protein ladder (PageRuler™ Prestained Protein Ladder, 10 to 180 kDa, # 26616), was used as size standards for monitoring protein migration, protein transfer to membranes, and sizing proteins. The blots were cut into 3 pieces at ~ 70 kDa, a little under ~ 55 kDa, and a little under ~ 40 kDa for MDM2 (90 kDa), p53 (53 kDa), and actin (42 kDa), respectively, prior to hybridization with antibodies. Monoclonal mouse anti-human primary antibodies Actin 1:1000 (#: C4: sc-47778, Santacruz Biotechnology, INC.), MDM2 1:300 (#: OP46-100UG, Merck Millipore), and p53 1:250 (#: 2B2.68: sc-71817, Santacruz Biotechnology, INC.) were used^71^. Secondary goat anti-mouse HRP-conjugated antibodies (#: STAR207P, BIO-RAD) were used at 1:5000. All antibodies were diluted in 5% milk/1XTBS-Tween (w/v). Enhanced chemiluminescence (GE Life Sciences, UK) and X-ray film (Fujifilm, India) were used to visualize the proteins. Image J software (National Institute of Health, USA) was used to quantify and analyze the intensity of visualized bands.

### Statistical analysis

The Kolmogorov–Smirnov test was used to assess the normality of the continuous variables. The Student unpaired t-test and the Mann–Whitney test were used for normally distributed and non-normally distributed variables respectively. The results were summarized as mean (± standard error of mean) if normally distributed or median (± quartile range, P25–P75) if not normally distributed. A Spearman correlation coefficient test was used to check for association between variables. All statistical analyses were performed in the SPSS 22.0 or GraphPad Prism 9; and significant differences are defined as *p* < 0*.*05.

### Ethics approval

The study was approved by the Ethics Committee of Royan Institute (IR.ACECR.ROYAN.R.EC.1401.027) and conducted in accordance with approved institutional guidelines.

## Supplementary Information

Supplementary Information.

## Data Availability

The data supporting the findings of this study are available within the paper and the Supplementary Information. The RNASeq data were downloaded from the Gene Expression Omnibus (GEO) database under accession numbers GSE138518 and GSE155489 which are available at the following URL: https://www.ncbi.nlm.nih.gov/geo/query/acc.cgi?acc=GSE138518 and https://www.ncbi.nlm.nih.gov/geo/query/acc.cgi?acc=GSE155489. The microarray data were downloaded from the GEO database under accession numbers GSE34526, GSE80432 and GSE10946 which are available at the following URL: https://www.ncbi.nlm.nih.gov/geo/query/acc.cgi?acc=GSE34526, https://www.ncbi.nlm.nih.gov/geo/query/acc.cgi?acc=GSE80432, and https://www.ncbi.nlm.nih.gov/geo/query/acc.cgi?acc=GSE10946.
