# Supplementary material for: Evaluation of the p53 pathway in polycystic ovarian syndrome pathogenesis and apoptosis enhancement in human granulosa cells through transcriptome data analysis
Source: Sci Rep. 2023 Jul 19;13:11648. doi: 10.1038/s41598-023-38340-1 (PMC10356838; doi:10.1038/s41598-023-38340-1)
Supplement: Supplementary file 1 — Supplementary Information. [file 41598_2023_38340_MOESM1_ESM.pdf]

**Supplementary Information**

# Evaluation of the p53 pathway in polycystic ovarian syndrome pathogenesis and apoptosis enhancement in human granulosa cells through transcriptome data analysis

**Zanjirband, M**., Hodayi, R., Safaeinejad, Z., Nasr-Esfahani, MH* & Ghaedi-Heydari, R**

**Mohammad Hossein Nasr-Esfahani: mh.nasr-esfahani@royaninstitute.org**

**(ORCID identifier: 0000-0003-1983-3435)**

**Maryam Zanjirband: maryamzanjirband118@gmail.com**

**(ORCID identifier: 0000-0002-5946-0998)**

**Raziyeh Hodayi: raziyeh.h1374@gmail.com**

**Zahra Safaeinejad, email: safayinejad@yahoo.com**

**Rasoul Ghaedi-Heydari, email: rasoulghaedi.heydari@gmail.com**

**Supplementary Table 1:** Transcriptome datasets from GCs employed in the current study.

| GEO Reference Series[a] | PCOS Subset | Control Subset | Platform |
|---|---|---|---|
| GSE34526 | 7 | 3 | Affymetrix Human Genome U133 Plus 2.0 |
| GSE138518 | 3 | 3 | Illumina HiSeq 2000 |
| GSE155489[b] | 4 | 4 | HiSeq X Ten |
| GSE80432[c] | 8 | 8 | Affymetrix Human Gene 1.0 ST |
| GSE10946[d] | 12 | 11 | Affymetrix Human Genome U133 Plus 2.0 |

a: Data were taken from the https://www.ncbi.nlm.nih.gov/geo/

b: Data related to the cumulus GCs in this study was used but those associated with oocytes were excluded.

c & d: According to PCA analysis, these datasets were removed from this study.

**Supplementary Table 2**: List of differentially expressed p53 target genes in PCOS granulosa cells: PCOS women versus control with $p < 0.05$ and $|\log FC| \geq 1$.

| GEO Reference | Ensemble Gene ID | Gene Symbol | Gene Name [a] |
|---|---|---|---|
| GSE34526 | ENSG00000015475 | *BID* | BH3 interacting domain death agonist |
| | ENSG00000087088 | *BAX* | BCL2 associated X |
| | ENSG00000171862 | *PTEN* | Phosphatase and tensin homolog |
| | ENSG00000064012 | *CASP8* | Caspase 8 |
| | ENSG00000026103 | *FAS* | Fas cell surface death receptor |
| | **ENSG00000134057** | ***CCNB1*** | **Cyclin B1** |
| | ENSG00000157456 | *CCNB2* | Cyclin B2 |
| | ENSG00000112576 | *CCND3* | Cyclin D3 |
| | ENSG00000175305 | *CCNE2* | Cyclin E2 |
| | ENSG00000113328 | *CCNG1* | Cyclin G1 |
| | ENSG00000175793 | *SFN* | Stratifin |
| | **ENSG00000135679** | ***MDM2*** | **Mouse double minute-2 homolog** |
| | ENSG00000137801 | *THBS1* | Thrombospondin 1 |
| | ENSG00000085117 | *CD82* | Cluster of differentiation 82 |
| | ENSG00000115107 | *STEAP3* | Six-transmembrane epithelial antigen of prostate 3 |
| GSE138518 | ENSG00000170312 | *CDK1* | Cyclin dependent kinase 1 |
| | ENSG00000134057 | *CCNB1* | Cyclin B1 |
| | **ENSG00000110092** | ***CCND1*** | **Cyclin D1** |
| | **ENSG00000181790** | ***ADGRB1*** | **Adhesion G protein-coupled receptor B1** |
| | **ENSG00000085117** | ***CD82*** | **Cluster of differentiation 82** |
| | ENSG00000141682 | *PMAIP1* | Phorbol-12-myristate-13-acetate-induced protein 1 |
| | ENSG00000026103 | *FAS* | Fas cell surface death receptor |
| GSE155489 | ENSG00000170312 | *CDK1* | Cyclin dependent kinase 1 |
| | ENSG00000112576 | *CCND3* | Cyclin D3 |
| | ENSG00000137801 | *THBS1* | Thrombospondin 1 |
| | ENSG00000141682 | *PMAIP1* | Phorbol-12-myristate-13-acetate-induced protein 1 |

| | | | |
|---|---|---|---|
| | ENSG00000130222 | *GADD45G* | Growth arrest and DNA damage inducible gamma |
| | **ENSG00000106366** | ***SERPINE1*** | **Serpin family E member 1** |

a: Down-regulated genes were shown as bold.

**Supplementary Table 3:** The list of significantly enriched KEGG pathways of associated PCOS related p53 downstream target genes which were differentially expressed.

| **Term** | **Overlap** | ***p-value*** | **Adjusted *p-value*** | **Genes** |
|---|---|---|---|---|
| p53 signaling pathway | 21/73 | *2.59E-66* | *2.90E-64* | *STEAP3; CD82; SERPINE1; PTEN; THBS1; CCNB2; CCND3 CCNB1; CASP8; CCND1; PMAIP1; SFN; GADD45G CCNE2; ADGRB1; CCNG1; CDK1; MDM2; FAS; BAX* |
| Cellular senescence | 11/156 | *1.51E-23* | *8.47E-22* | *SERPINE1; PTEN; GADD45G; CCNB2 CCND3; CCNB1; CND2; CCND1; CCNE2; DK1; MDM2* |
| Cell cycle | 10/124 | *1.68E-20* | *6.26E-19* | *CCNB2; CCND3; CCNB1; CCND2; CCND1; CCNE2 CDK1; MDM2; SFN; GADD45G* |
| FoxO signaling pathway | 7/131 | *3.86E-12* | *4.32E-11* | *CCNB2; CCNB1; CCND2; CCND1; PTEN; MDM2; GADD45G* |
| Apoptosis | 6/142 | *4.60E-10* | *3.68E-09* | *CASP8; PMAIP1; FAS; BAX; BID; GADD45G* |
| PI3K-Akt signaling pathway | 7/354 | *1.05E-08* | *6.72E-08* | *CCND3; CCND2; CCND1; CCNE2; PTEN; MDM2; THBS1* |
| Focal adhesion | 5/201 | *5.40E-06* | *1.78E-05* | *CCND3; CCND2; CCND1; PTEN; THBS1* |
| AGE-RAGE signaling pathway in diabetic complications | 4/100 | *8.08E-06* | *2.51E-05* | *CCND1; CDK4; SERPINE1; BAX* |
| Progesterone-mediated oocyte maturation | 3/100 | *2.90E-04* | *7.22E-04* | *CCNB2; CCNB1; CDK1* |

**Supplementary Table 4:** The primers and their sequences were used for qRT-PCR experiments. F, Forward; R, Reverse.

| | |
|---|---|
| *FAS* | F- AGATTGTGTGATGAAGGACATGG |
| | R- TGTTGCTGGTGAGTGTGCATT |
| *MDM2* | F- AGTAGCAGTGAATCTACAGGGA |
| | R- CTGATCCAACCAATCACCTGAAT |
| *PMAIP1* | F -GGAGGTGCACGTTTCATCAATTT |
| | R - AGGGTCTTCCATTCTTGCTTTAA |

**Supplementary Figure 1**

**a: GSE10946**

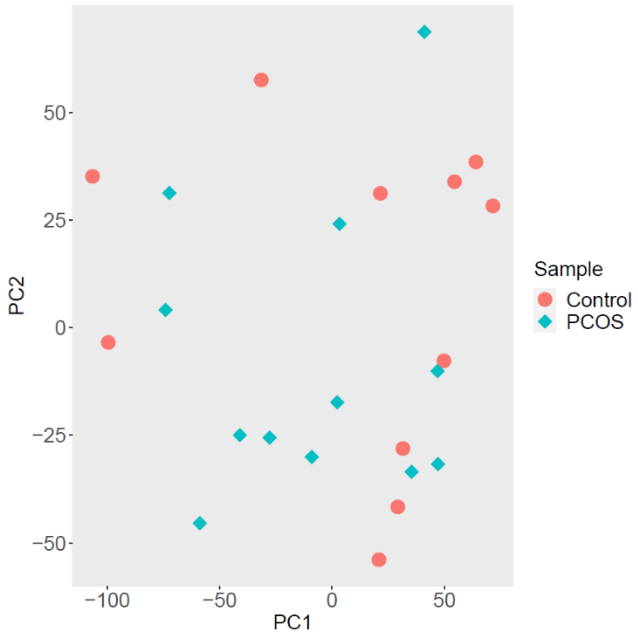


**b: GSE80432**

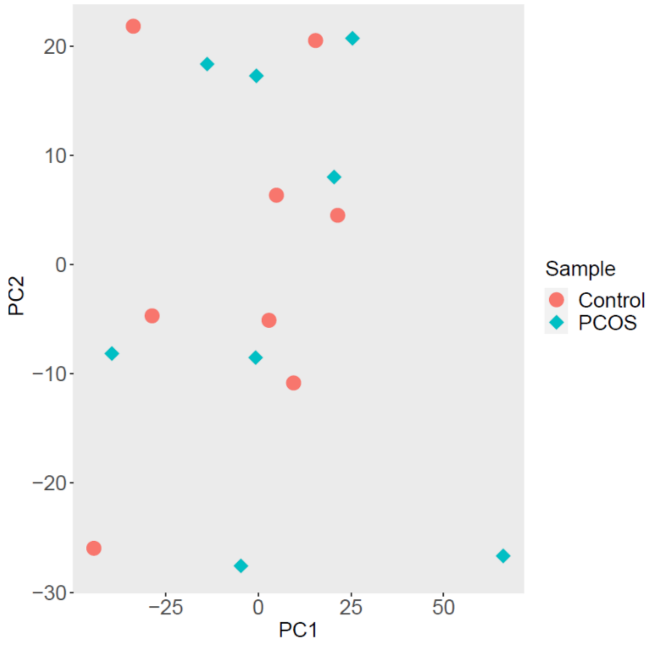


**Supplementary Figure 1:** PCA (Principal component analysis) plots were constructed by using a normalized gene expression matrix. (A) GSE10946 dataset, (B) GSE80432 dataset. PCA of transcriptome in GCs obtained from PCOS and control cases. The samples are represented by different colours as indicated in the right.

**Supplementary Figure 2**

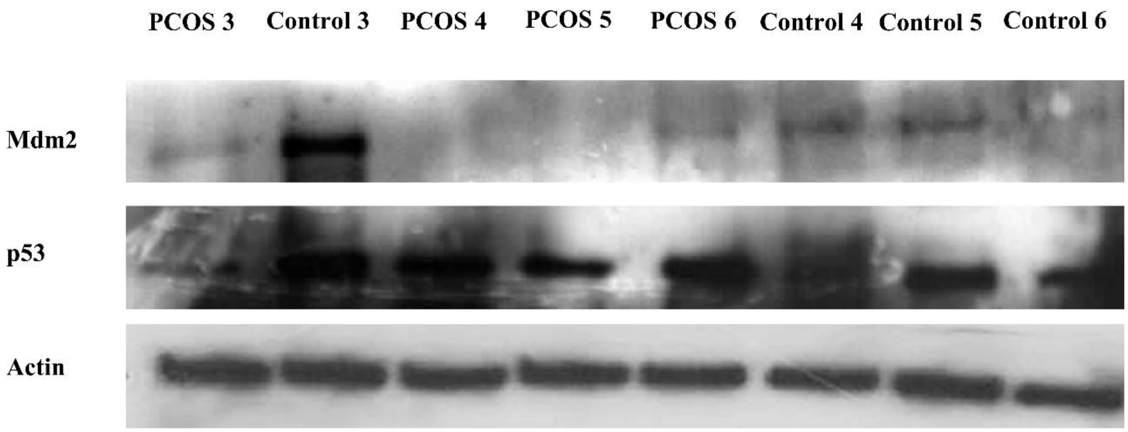


**Supplementary Figure 2:** Western blot analysis of p53 and Mdm2 proteins in PCOS women (PCOS 3-6) compared to the controls (Controls 3-6). The Thermo scientific protein ladder, (10 to 180 kDa) was used as size standards for monitoring protein migration, protein transfer to membranes, and sizing proteins. The blots were cut into 3 pieces at ~ 70 kDa, a little under ~ 55 kDa, and a little under ~ 40 kDa for Mdm2 (90 kDa), p53 (53 kDa), and actin (42 kDa), respectively, prior to hybridization with antibodies.

## Supplementary Figure 3

L: Deleted p53 cell line, B & a: control women (non-PCOS) C & d: PCOS women
N (0.5 RG7388), CC, & N (0.25 RG7388): 3 positive control cell line for p53, Con: Control

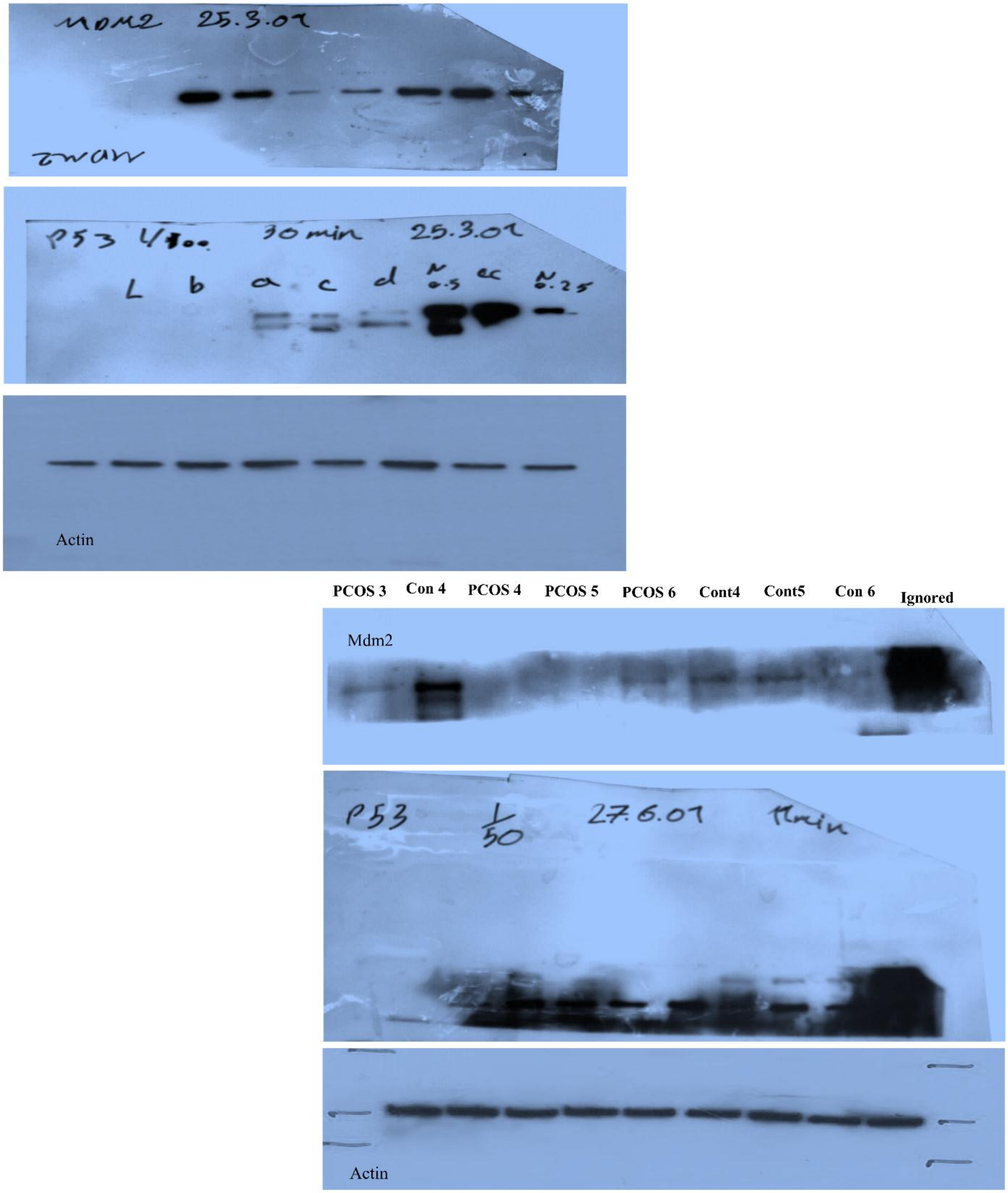


**Supplementary Figure 3:** The original unedited blots presenting Mdm2 (90 kDa), p53 (53 kDa), and actin (42 kDa) in Figure 5B and Supplementary Figure 2.
